# Relationship among subjective exercise experience, exercise behavior, and trait anxiety in adolescents

**DOI:** 10.1186/s12889-023-16536-4

**Published:** 2023-08-31

**Authors:** Shuyu Luo, Lian Feng, Jiabao Zhao

**Affiliations:** 1 Chongqing Institute of Foreign Studies, Chongqing, 401120 China; 2 Chongqing Youyang No.2 Middle School, Chongqing, 409899 China; 3 Faculty of Physical Education, China West Normal University, Nanchong, 637009 China

**Keywords:** Adolescents, Subjective exercise experience, Exercise behavior, Trait anxiety, Mediating effect

## Abstract

**Objective:**

To investigate the influence of subjective exercise experience on adolescent trait anxiety and to reveal the mediating effect of exercise behavior.

**Methods:**

Using the Subjective Exercise Experience Scale (SEES), Physical Exercise Rating Scale (PARS-3), and Trait Anxiety Inventory (T-AI), a questionnaire survey was conducted among 500 adolescents in Southwest China, and the SPSS21.0 and AMOS21.0 statistical analysis software was used to statistics and analysis on the questionnaires.

**Results:**

1) Among adolescents, the exercise behavior of boys was significantly higher than that of girls (*p* < 0.05), and the subjective exercise experience of students aged 9 to 12 was significantly higher than that of students aged 12 to 15 (*p* < 0.05). 2) The subjective exercise experience could directly and positively predict exercise behavior (β = 0.45, *p* < 0.001) and negatively predict trait anxiety (β = -0.26, *p* < 0.05), and exercise behavior could directly and negatively predict trait anxiety (β = -0.32, *p* < 0.01). 3) The exercise behavior played a partial mediating effect between subjective exercise experience and trait anxiety (the mediation effect was -0.14). Among them, compared with low- and high-exercise amounts, the exercise behavior of moderate exercise amounts had the strongest mediating effect between subjective exercise experience and trait anxiety.

**Conclusion:**

The good subjective exercise experience not only has direct benefits for improving trait anxiety in adolescents but also helps to improve their exercise behavior, enrich daily physical exercise activities, and indirectly promote the reduction of trait anxiety.

## Introduction

With the increasing pressure and academic burden of further studies, psychological problems such as anxiety among adolescents have gradually become the focus of social attention. According to statistics, about 1/3 of adolescents will suffer from anxiety disorders before adulthood, which can easily lead to poor academic performance, communication barriers, and other adverse consequences, and will affect their psychological and social functions [1, 2]. Anxiety in adolescents mainly refers to physical symptoms of physiological tension and anxiety, tension, and irritability [3, 4]. Among them, the trait anxiety refers to an individual’s tendency to evaluate internal stimuli or external events in a way that causes anxiety [5] and was a relatively stable personality trait or trait with individual differences [6], its effects on adolescents’ emotional state, self-control, and academic performance may be more lasting [7]. Studies have shown that people with high trait anxiety were more likely to experience high-intensity tension and anxiety in stressful situations [8], and there was a certain processing bias in cognitive processes such as attention, memory, and understanding, which easily damages their cognitive ability and behavioral performance [9, 10]. Adolescents with high trait anxiety will overestimate the risk of academic failure, easily feel helpless, couldn’t reasonably cope with academic pressure, and were difficult to achieve high academic performance [11]. Therefore, how to regulate or improve their trait anxiety was crucial for promoting adolescents’ mental health and academic performance.

For a long time, the influence of physical exercise on anxiety and other negative psychological emotions has attracted the attention of many scholars [12–14]. Compared with group psychological counseling, drug therapy, and behavioral intervention, physical exercise was considered to be the greenest, most economical, and environmentally friendly non-drug therapy to improve trait anxiety in adolescents. For example, studies have shown that different forms of physical activity could be effective in improving anxiety in individuals [15, 16], and it was accompanied by the improvement of executive function and emotion regulation ability [17], and exercise behaviors in different situations could improve the anxiety of individuals [18]. In addition, Fang [19] found through a survey of college students that different physical exercise participation has significant benefits on trait anxiety, and the duration of exercise, exercise intensity, and single exercise time all have varying degrees of influence on individual trait anxiety. Studies have also pointed out that moderate-intensity physical activity has the best effect on improving individual mental health [20, 21]. The results suggest that there may be a “dose effect” on individual trait anxiety with different amounts of physical activity.

The social learning theory holds that experiences or experiences in specific situations could change behavioral perceptions, and determine behavioral decision-making and expression [22, 23]. In the process of exercise, subjective exercise experience was closely related to individual exercise behavior [24]. On the one hand, the subjective experience was a cognitive operation experience [25]. When an individual has a positive experience in past exercise activities, there will be a strong desire to exercise, accompanied by a desire to practice repeatedly; however, those who lack positive experience will have established and consistent cognitive responses in exercise activities, and often show negative tendencies such as rejection and withdrawal and even restrict exercise behavior [26]. On the other hand, the subjective exercise experience was a state of behavioral fluency [27], which can not only affect the individual’s input state but also the basis for decision-making of exercise behavior [28]. The positive exercise experience will fill people with a sense of pleasure and availability, help to stimulate young people’s exercise awareness and commitment, and help young people to clarify exercise intentions and strengthen their commitment to exercise [29]. Accordingly, it can be speculated that a good subjective exercise experience helps to promote the increase of individual exercise behavior. In addition, there was an association between subjective exercise experience and exercisers’ mental health. Wang [30] found through structural equation modeling that the subjective exercise experience of adolescents was significantly correlated with depression tendency, manifested as the improvement of positive well-being, or the weakening of psychological distress and fatigue, which could effectively improve the depression of adolescents. Similarly, studies have found that the increase in positive well-being and the reduction of mental fatigue during physical activity could effectively weaken the individual’s negative emotions such as anxiety and depression [31]. Similar findings were also found in athletes, that is, sports mental state, general self-efficacy, mood, and other mental states are closely related to competition anxiety [32], but there were few studies on trait anxiety in adolescents.

To sum up, there were different degrees of correlation among individuals’ subjective exercise experience, exercise behavior, and trait anxiety. However, in adolescents, some key issues such as the effect of subjective exercise experience on their trait anxiety and the role of exercise behavior in it remain unclear. Given this, this study proposes the following hypotheses: 1) the subjective exercise experience has a direct predictive effect on trait anxiety in adolescents; 2) exercise behavior has a direct predictive effect on trait anxiety; 3) exercise behavior has a mediating effect between subjective exercise experience and trait anxiety; 4) there was a “dose effect” of exercise amount in the mediating effect of exercise behavior, and the mediating effect of exercise behavior with moderate exercise amount was the best.

## Participants and methods

### Participants

This study adopts a cross-sectional survey research design. First, the cluster random sampling method was used to sample the primary and secondary schools in southwest China (Chongqing City and Liupanshui City) according to a ratio of about 1:80, and a total of six schools were randomly selected, among which three primary and secondary schools in Chongqing and Liupanshui were selected. Secondly, according to the actual needs of the research, this study conducted a cluster random sampling of students in the selected schools according to the ratio of about 1:100 with student numbers and selected 80 to 100 people in each school for the questionnaire survey. The selected students will be informed to participate in this research and will be assigned to the designated classroom to complete the questionnaire. To ensure the authenticity of the questionnaires, we fully explained the contents and precautions of the questionnaires to the participants before issuing the questionnaires and adopted the method of distributing and returning the questionnaires on-site, and the participants needed to spend 15 min in class to complete the questionnaires. Corresponding data indicators are obtained by conducting questionnaire surveys on the respondents, and relevant measurement and prediction models were used to compare the research objectives. For this, a single evaluation was carried out for data collection, which was subsequently statistically processed. A total of 500 questionnaires were distributed in this study, and a total of 482 were recovered. Through screening (exclusion principle: 25% missing questions, regular answers or obvious questions, et al.), 450 valid questionnaires were determined (the effective rate was 93.36%). Among them, there were 256 boys and 194 girls, the average age was (12.36 ± 3.28) years, and the average BMI was (19.55 ± 4.76).

### Measuring tools

#### Subjective exercise experience scale (SEES)

Two subscales of “positive well-being” and “psychological distress” in the SEES compiled and revised by Mcauley et al. [33] were selected (each containing 4 items, a total of 8 items), using Likert 5-point scoring, according to the options “completely incompatible ~ completely in line” as 1 to 5 points respectively. Among them, “psychological distress” adopts reverse scoring, and the total score of SEES was the sum of the scores of each item of the two subscales, and a higher score indicates a better subjective exercise experience. After the test–retest, it was found that the item loads of the positive well-being and psychological distress scales were all between 0.50 and 0.95, the combined reliability (CR) was greater than 0.60, and the average variance extraction value (AVE) was also greater than 0.50. It shows that the convergent validity of the two scales was at a good level, and the AVE values of each scale were all greater than the square value of the correlation coefficient, indicating that they have good discriminant validity. After the internal consistency test, the Cronbach α coefficient of positive well-being was 0.87, the Cronbach α coefficient of psychological distress was 0.81, and the overall Cronbach α coefficient of the total scale was 0.84. The confirmatory factor analysis was performed on the scale, and the results of measurement model validation: x^2^/df = 1.98, TLI = 0.98, CFI = 0.96, AGFI = 0.96, IFI = 0.93, RMSEA = 0.03. It shows that the scale has good construct validity and reliability.

#### Physical activity rating scale (PARS-3)

Adopted and revised Liang’s [34] Physical Activity Rating Scale, which was evaluated from three aspects of exercise intensity, exercise frequency, and exercise time. The 5-point Likert method was used for quantification, the exercise intensity and frequency were scored from 1 to 5 on a scale of 1 to 5, the exercise time was scored from 0 to 4 on a scale of 1 to 5, and the formula “exercise intensity × exercise time × exercise frequency” was used to quantify the total score of the subjects’ exercise behavior (the highest score was 100 points, and the lowest score was 0 points), and a higher score means a greater amount of exercise. Meanwhile, the evaluation criteria for exercise amount were: the low exercise amount ≤ 19 points, moderate exercise amount was 20 to 42 points, and high exercise amount ≥ 43 points. The test–retest reliability of the scale was high, and the correlation coefficient r = 0.82.

#### Trait anxiety inventory (T-AI)

The state-trait anxiety inventory (STAI) was compiled and revised by Spielberger [8], and this study only used the Trait Anxiety Inventory (T-AI). The scale was a one-dimensional scale with a total of 20 items, including 11 items for forwarding scoring and 9 items for reverse scoring. A 4-point Likert score was used, and the options “not at all to very consistent” were counted as 1 to 4 points, with higher scores indicating higher levels of trait anxiety. After the test–retest, it was found that the item loads of the Trait Anxiety Scale were all between 0.50 and 0.95, the combined reliability (CR) was greater than 0.60, and the average variance extraction value (AVE) was also greater than 0.50. It shows that the scale has good convergent validity, and the AVE value was greater than the square value of the correlation coefficient, indicating that the scale has good discriminant validity. After the internal consistency test, the overall Cronbach α coefficient of the scale was 0.83. The confirmatory factor analysis was performed on the scale to measure the model validation results: x^2^/df = 2.57, TLI = 0.97, CFI = 0.95, AGFI = 0.97, IFI = 0.96, RMSEA = 0.04. It shows that the scale has good construct validity and reliability.

### Mathematical statistics

In this study, SPSS21.0 was used to process and analyze the data, and factor analysis, and internal consistency tests were used to examine the reliability and validity of the scale. Descriptive analysis such as descriptive statistics and independent sample t-test was used to investigate the current differences in adolescents’ subjective exercise experience, exercise behavior, and trait anxiety. The Pearson correlation analysis was used to test the correlation coefficient between variables (r), the general linear regression analysis was used to investigate the degree of influence of independent variables on dependent variables, expressed as standardized coefficients (β), and the Bootstrap analysis and AMOS21.0 software were used to establish a structural equation model to investigate the relationship between variables and the mediating effect of exercise behavior. The significance level of all indicators was set at *p* < 0.05.

## Results

### Common method bias test

This study used a questionnaire survey method, and all questionnaire items were filled out by the subjects themselves, so there may be a common method bias in the measurement. To minimize the impact of common method bias on the results, this study used procedures control methods such as anonymous questionnaire measurement and standardized test administration to control it accordingly. After data collection, this study also used Harman’s one-factor test to examine the problem of common method bias [35]. Specifically, the measurement items of all variables were put together for unrotated factor analysis. The results show that there were 7 factors with characteristic roots greater than 1, and the variance explained by the first factor was 28.78%, which was less than the critical standard of 40%. It can be seen that the common method bias did not cause serious problems in this study.

### Demographic differences analysis

It could be seen from Table 1, there was no significant gender difference in subjective exercise experience (t = 2.15, *p* > 0.05) and trait anxiety (t = 1.19, *p* > 0.05), but boys’ exercise behavior was significantly higher in female students (t = 3.99, *p* < 0.05). Subjects aged 9 to 12 had significantly higher subjective exercise experience than those aged 12 to 15 (t = 3.85, *p* < 0.05), but students’ exercise behavior (t = 2.07, *p* > 0.05) and trait anxiety (t = 1.01, *p* > 0.05), there was no significant age difference.

**Table 1:** Demographic differences analysis of subjective exercise experience, exercise behavior, and trait anxiety among adolescents

Category	Variable	Subjective exercise experience	Exercise behavior	Trait anxiety
Gender	Men	28.56 ± 3.23	25.12 ± 5.32	41.36 ± 4.56
	Women	26.18 ± 3.10	21.88 ± 4.80	42.15 ± 4.71
	t	2.15	3.99^*^	1.19
	*p*	0.17	0.02	0.91
Age	9 to 12	29.44 ± 3.78	24.22 ± 5.18	41.62 ± 4.37
	12 to 15	24.65 ± 2.86	22.70 ± 4.54	41.89 ± 5.11
	t	3.85^*^	2.07	1.01
	*p*	0.03	0.28	0.95

^*^p < 0.05

### Correlation analysis of subjective exercise experience, exercise behavior, and trait anxiety

The correlation analysis showed (Table 2) that the subjective exercise experience of adolescents was significantly positively correlated with exercise behavior (r = 0.45, *p* < 0.001), and the subjective exercise experience was significantly negatively correlated with trait anxiety (r = -0.26, *p* < 0.05), the exercise behavior was significantly negatively correlated with trait anxiety (r = -0.32, *p* < 0.01). The correlations between the main variables reached a significant level, which provided a good basis for the subsequent test of the mediation effect.

**Table 2:** Correlation analysis of subjective exercise experience, exercise behavior, and trait anxiety

Variable	M ± SD	Subjective exercise experience	Exercise behavior	Trait anxiety
Subjective exercise experience	27.53 ± 3.19	1	1	1
Exercise behavior	23.56 ± 4.69	0.45^***^		
Trait anxiety	42.02 ± 5.11	-0.26^*^	-0.32^**^	

^*^p < 0.05, ^**^p < 0.01, ^***^p < 0.001

## Effect and path relationship of subjective exercise experience on trait anxiety

### Direct effect analysis

The linear regression analysis was used to examine the direct relationship between the variables (Table 3). First, after controlling for demographic variables such as gender and age, this study used subjective exercise experience as an independent variable, and exercise behavior and trait anxiety as dependent variables, respectively. The results showed that subjective exercise experience could positively predict individual exercise behavior (β = 0.45, *p* < 0.001), which could explain 20% of the variance. The subjective exercise experience negatively predicted individual trait anxiety (β = -0.26, *p* < 0.05), explaining 7% of the variance. Second, exercise behavior was used as an independent variable, and trait anxiety was used as a dependent variable. The results showed that exercise behavior could negatively predict individual trait anxiety (β = -0.32, *p* < 0.01), which could explain 10% of the variance.

**Table 3:** Linear regression analysis of subjective exercise experience, exercise behavior, and trait anxiety

Variable	Exercise behavior			Trait anxiety		
	β	R^2^	95%CI	β	R^2^	95%CI
Subjective exercise experience	0.45^***^	0.20	(0.41,0.47)	-0.26^*^	0.07	(-0.28,-0.23)
Exercise behavior	—	—	—	-0.32^**^	0.10	(-0.33,-0.29)

^*^p < 0.05, ^**^p < 0.01, ^***^p < 0.001

#### Mediation effect analysis

The mediation effect test method of Baron and Kenny [36] was adopted, which mainly includes three steps: first, the independent variable has an influence on the dependent variable, and the regression coefficient reaches a significant level. Second, the independent variable influences the intermediary variable, and the regression coefficient was significant. Third, the common influence of independent and mediating variables on the dependent variable was significant, among them, the influence of the mediator variable on the dependent variable must reach a significant level. At this time, if the influence of the independent variable on the dependent variable becomes insignificant, the mediator variable plays a complete mediating role, and if the independent variable’s influence on the dependent variable decreases, the mediator variable plays a partial mediating role.

**Fig. 1:**
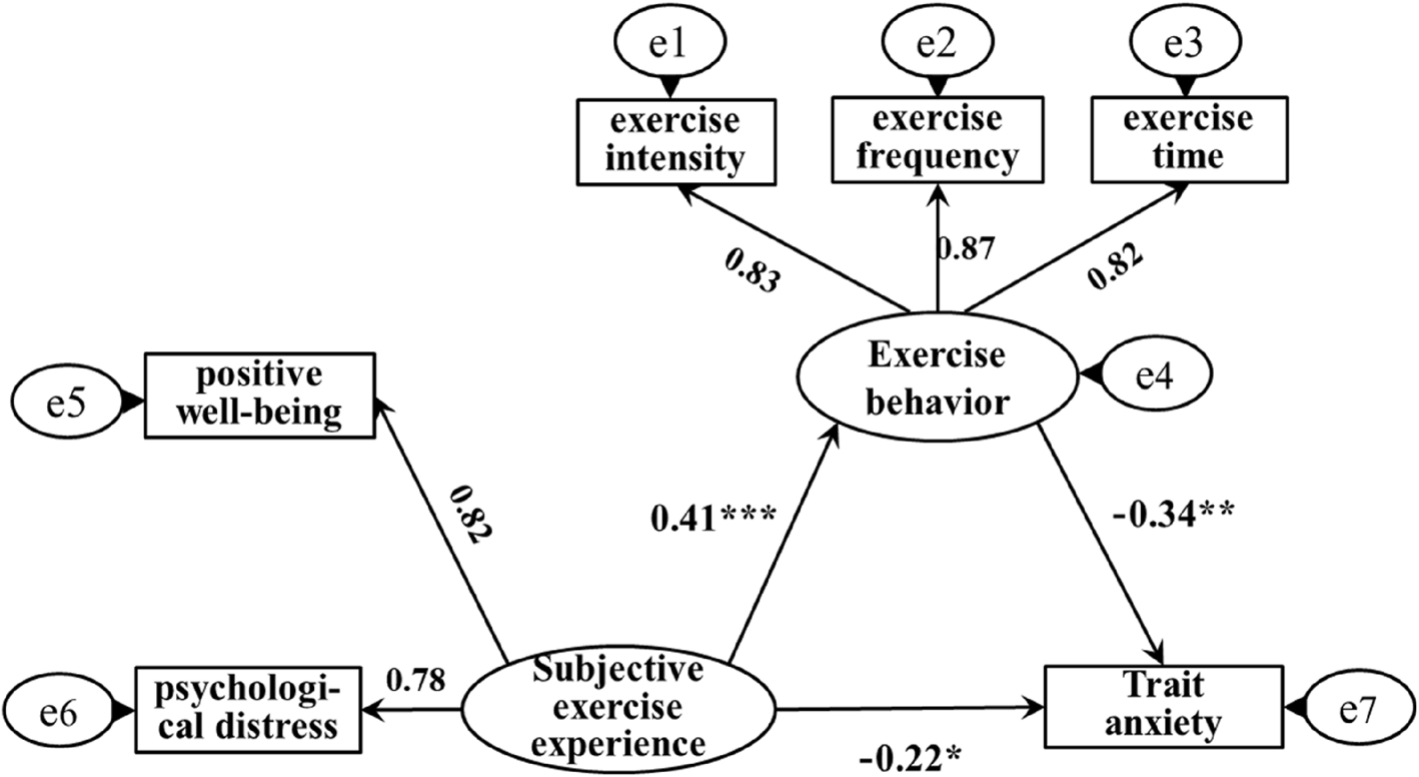
Mediating effect model of exercise behavior between subjective exercise experience and trait anxiety. Note: ^*^p < 0.05, ^**^p < 0.01, ^***^p < 0.001

The AMOS software was used to establish a structural equation model to investigate the mediating effect of exercise behavior between subjective exercise experience and trait anxiety (please see Fig. 1). The fitting indicators of the model were: x^2^/df = 1.79, RMSEA = 0.03, GFI = 0.97, TLI = 0.98, NFI = 0.95, IFI = 0.93, AGFI = 0.95, it shows that the model has a good degree of fit, indicating that it was suitable for the mediation effect test. The results of this study showed that the path coefficient of subjective exercise experience on trait anxiety was significant (β1 = -0.26, SE = 0.03, *p* < 0.05). After adding exercise behavior as a mediating variable, the path coefficient of subjective exercise experience to exercise behavior was significant (β = 0.41, SE = 0.02, *p* < 0.001), and the path coefficient of exercise behavior to trait anxiety was significant (β = -0.34, SE = 0.02, *p* < 0.01). However, the path coefficient of subjective exercise experience on trait anxiety decreased, but still showed a significant level (β2 = -0.22, SE = 0.04, *p* < 0.05), indicating that exercise behavior partially mediates the relationship between subjective exercise experience and trait anxiety effect. The effect decomposition of each path was shown in Table 4. Therefore, the hypotheses H1, H2, and H3 of this study were all confirmed.

**Table 4:** Decomposition of path effects of subjective exercise experience on trait anxiety

Influence path	Standardized effect size	The ratio of the total effect	Bootstrap SE	Significance
Total effect	-0.36	100%	0.03	significant
Direct effect	-0.22	61.11%	0.02	significant
Total mediation effect	-0.14	38.89%	0.04	significant
Subjective exercise experience → Exercise behavior → Trait anxiety	0.41 × (-0.34) = -0.14	38.89%	0.04	significant

The above studies showed that exercise behavior plays a partial mediating role between subjective exercise experience and trait anxiety, but exercise behavior was a comprehensive variable, and there was often a “dose effect” of exercise amount. Fang et al. [37] found that the bias-corrected percentile Bootstrap Method (Bia-corrected Bootstrap Method) was more powerful than the traditional Sobel test when doing the significance test of the mediating effect. The basic idea of this method was to do repeated sampling with replacement in the original data and extract equal sample data to test the mediation effect. Given this, to further investigate the differences in the effect of different amounts of physical exercise on improving trait anxiety in adolescents, Model 4 of the SPSS macro compiled by Hayes [38] (http://www.afhayes.com) was used to estimate the 95% confidence interval of the mediation effect by extracting 5,000 samples and to test the mediation effect of different physical exercise amounts. If the 95% confidence interval of the mediation effect does not include 0, it means that the mediation effect was significant; otherwise, it means that the mediation effect was not significant. The mediation effect test in this study was conducted under the control of statistical variables such as gender and age.

The results of regression analysis in this study showed (Tables 5, 6, and 7): 1) The subjective exercise experience could significantly and positively predict low exercise amount (β = 0.30, *p* < 0.01), and low exercise amount could significantly negatively predict trait anxiety (β = -0.23, *p* < 0.05) when both subjective exercise experience and low exercise amount predicted trait anxiety, the subjective exercise experience significantly and negatively predicted the trait anxiety (β = -0.19, *p* < 0.05). 2) The subjective exercise experience could significantly and positively predict moderate exercise amount (β = 0.43, *p* < 0.001), and moderate exercise amount could significantly and negatively predict the trait anxiety (β = -0.36, *p* < 0.001) when both subjective exercise experience and moderate exercise amount predicted the trait anxiety, the subjective exercise experience significantly and negatively predicted trait anxiety (β = -0.21, *p* < 0.05). 3) The subjective exercise experience could significantly and positively predict high exercise amount (β = 0.40, *p* < 0.001), and the high exercise amount could significantly and negatively predict the trait anxiety (β = -0.20, *p* < 0.05) when both subjective exercise experience and high exercise amount predicted trait anxiety, the subjective exercise experience significantly and negatively predicted trait anxiety (β = -0.19, *p* < 0.05).

**Table 5:** Mediating effect of low exercise amount between subjective exercise experience and trait anxiety

Variable	Low exercise amount			Trait anxiety		
	t	β	95%CI	t	β	95%CI
Subjective exercise experience	2.78	0.30^**^	(0.25,0.32)	-2.17	-0.19^*^	(-0.23,-0.14)
Low exercise amount				-2.08	-0.23^*^	(-0.26,-0.21)
R^2^	0.09			0.11		
F	4.14^**^			4.02^*^		

^*^p < 0.05, ^**^p < 0.01

**Table 6:** Mediating effect of moderate exercise amount between subjective exercise experience and trait anxiety

Variable	Moderate exercise amount			Trait anxiety		
	t	β	95%CI	t	β	95%CI
Subjective exercise experience	4.15	0.43^***^	(0.39,0.47)	-2.33	-0.21^*^	(-0.23,-0.14)
Moderate exercise amount				-4.01	-0.36^***^	(-0.41,-0.33)
R^2^	0.18			0.20		
F	5.22^***^			6.16^***^		

^*^p < 0.05, ^***^p < 0.001

**Table 7:** Mediating effect of high exercise amount between subjective exercise experience and trait anxiety

Variable	High exercise amount			Trait anxiety		
	t	β	95%CI	t	β	95%CI
Subjective exercise experience	4.03	0.40^***^	(0.35,0.42)	-2.16	-0.19^*^	(-0.21,-0.15)
High exercise amount				-2.01	-0.20^*^	(-0.23,-0.18)
R^2^	0.16			0.12		
F	5.03^***^			3.97^*^		

^*^p < 0.05, ^***^p < 0.001

**Fig. 2:**
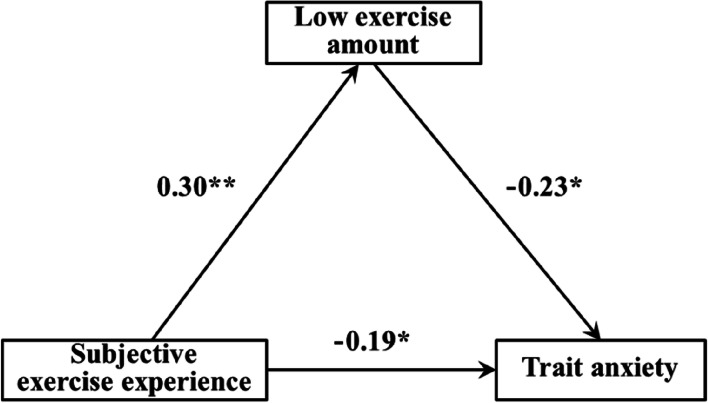
Mediating model of low exercise amount between subjective exercise experience and trait anxiety

**Fig. 3:**
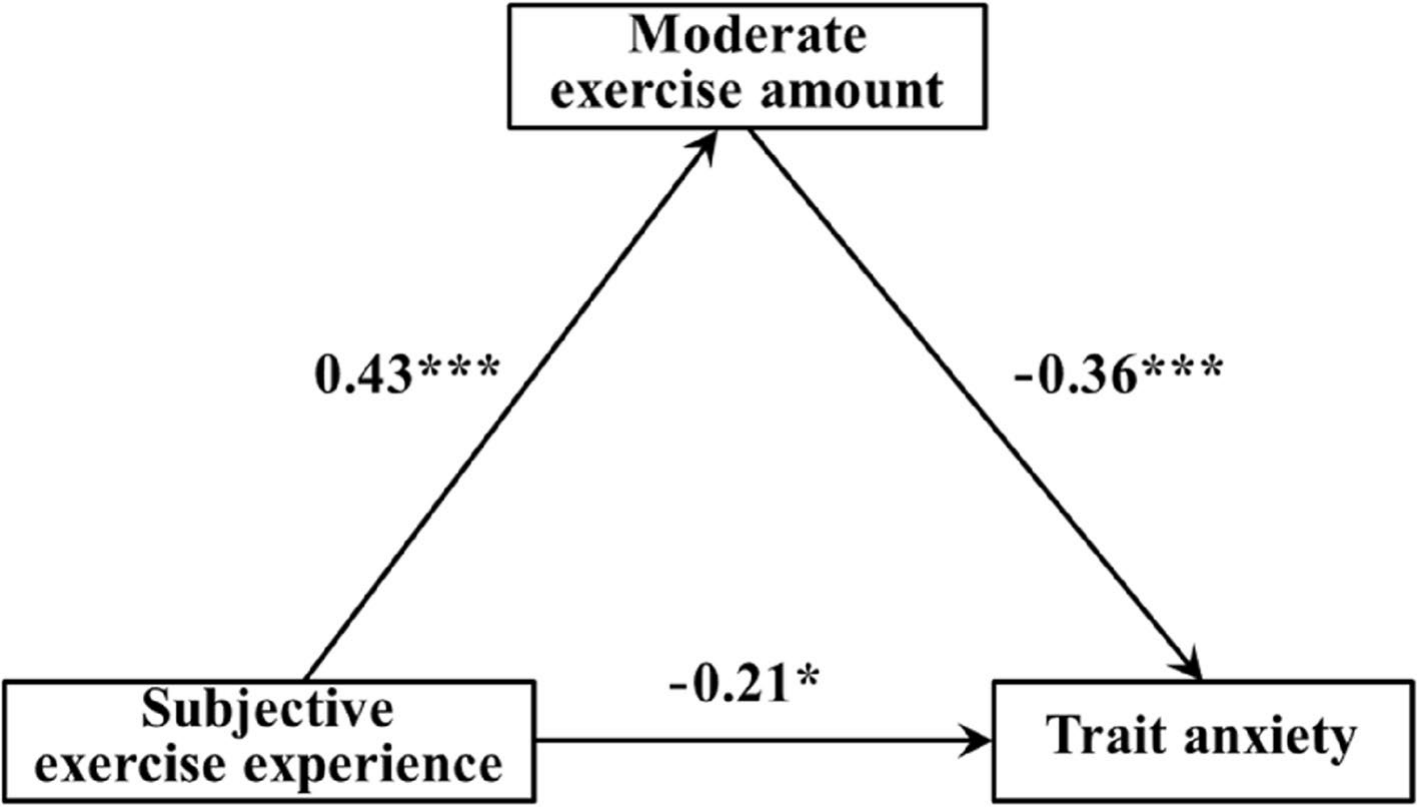
Mediating model of moderate exercise amount between subjective exercise experience and trait anxiety

**Fig. 4:**
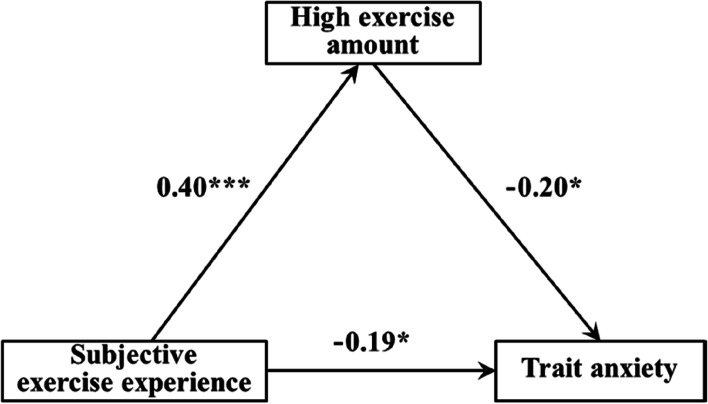
Mediating model of high exercise amount between subjective exercise experience and trait anxiety

Figures 2, 3 and 4 showed the path model and path coefficient values of the relationship between subjective exercise experience and trait anxiety under low, moderate, and high exercise amounts, respectively. In this study, there were three valid mediation effect models: 1) “Subjective exercise experience → low exercise amount → trait anxiety”, the confidence interval of this path does not contain 0, indicating that the low exercise amount has a significant mediating effect between subjective exercise experience and trait anxiety (standardized effect value: 0.30 × (-0.23) = -0.07, accounting for 26.92% of the total effect), and the direct path coefficient of subjective exercise experience to trait anxiety was significant, indicating that the low exercise amount played a partial mediating role in this model. 2) “Subjective exercise experience → moderate exercise amount → trait anxiety”, the confidence interval of this path does not contain 0, indicating that the moderate exercise amount has a significant mediating effect between subjective exercise experience and trait anxiety (standardized effect value: 0.43 × (-0.36) = -0.15, accounting for 41.67% of the total effect), and the direct path coefficient of subjective exercise experience to trait anxiety was significant, indicating that the moderate exercise amount plays a partial mediating role in this model. 3) “Subjective exercise experience → high exercise amount → trait anxiety”, the confidence interval of this path does not contain 0, indicating that the high exercise amount has a significant mediating effect between subjective exercise experience and trait anxiety (standardized effect value: 0.40 × (-0.20) = -0.08, accounting for 29.63% of the total effect), and the direct path coefficient of subjective exercise experience to trait anxiety was significant, indicating that the high exercise amount played a partial mediating role in this model. Therefore, hypothesis H4 of this study was confirmed.

## Discussion

### Demographic difference analysis

This study found that there were no significant gender differences in subjective exercise experience and trait anxiety among adolescents, which indicates that boys and girls have the same cognitive feelings in previous exercise experiences, and both can obtain similar positive exercise experiences such as pleasure, happiness, and satisfaction through physical exercise, or negative exercise experiences such as fatigue and boredom. This was different from previous studies [39], and we speculate that this difference may be due to the different regions, environments, and personality traits of the respondents. Meanwhile, both boys and girls shoulder higher expectations from parents and teachers and face the same academic pressure or growth pressure, so they were prone to negative emotions such as trait anxiety. Interestingly, the exercise behavior of boys was significantly higher than that of girls. Research shows that compared with girls, boys will maintain relatively independent, stable, and regular exercise habits in their spare time due to their stronger motivation and desire to exercise [40], thereby increasing exercise behaviors. This study also found that the subjective exercise experience of primary school students (9 to 12 years old) was significantly higher than that of junior high school students (12 to 15 years old). The primary school stage was in the critical stage of the rapid development of body movement perception ability and has an intuitive feeling for the experience or experience of exercise, so it was easier to have a higher subjective exercise experience. Subjective exercise experience is usually associated with exercise behavior, and a good exercise experience helps adolescents to obtain emotional feelings such as pleasure and happiness and to form exercise habits or behaviors [29, 39] and mental health has a positive promoting effect. However, this study found no significant age differences in exercise behavior and trait anxiety among adolescents of different ages. We speculate that this may be because primary school students are not good at converting good exercise experience into corresponding exercise behavior, and lack good exercise persistence and exercise decision-making power. Meanwhile, no matter whether in primary school or junior high school, they all face the same academic pressure such as college entrance [41], which leads to trait anxiety becoming a more common phenomenon among teenagers.

However, there was no significant age difference in exercise behavior and trait anxiety. On the one hand, it shows that primary school students were not good at converting good exercise experience into corresponding exercise behavior, and lack good exercise persistence and exercise decision-making ability. On the other hand, it reflects that both primary and junior high schools were facing the same academic pressures such as going to school, which leads to trait anxiety becoming a more common phenomenon in adolescents.

### The direct effect of subjective exercise experience on trait anxiety

In this study, the subjective exercise experience of adolescents is closely related to trait anxiety, and the former could directly and negatively predict trait anxiety, which means that a higher subjective exercise experience could effectively reduce individual trait anxiety. Previous studies have paid less attention to the direct effect of subjective exercise experience on trait anxiety, and only some studies have pointed out that a good subjective exercise experience has a direct relationship with psychological variables such as individual physical self-esteem [42] and exercise commitment [24]. Some studies have also shown that the acquisition of positive well-being after exercise could inhibit the generation of psychological distress and fatigue and have a certain positive effect on improving the depression tendency of adolescents [30]. It should be noted that subjective exercise experience was an individual’s subjective perception of physical and mental feelings after previous physical exercise [42], and the two dimensions of positive well-being and psychological distress it contains were bound to be directly related to mental health. We believe that higher positive well-being and lower psychological distress can effectively improve trait anxiety in adolescents, mainly through good exercise perception and experience ability to enrich the emotional experience, and then rationally and positively evaluate the anxiety experience tendency of internal stimuli or external events. In the past physical exercise experience, the generation of positive well-being and the reduction of psychological troubles and fatigue have a significant effect on enhancing the positive emotions of adolescents and reducing related negative emotions such as anxiety and depression [31, 43]. It should be pointed out that the direct effect of subjective exercise experience on trait anxiety in adolescents was also applicable in low, moderate, or high exercise volume models, suggesting that a good subjective exercise experience may be an independent variable for improving trait anxiety.

### The mediating effect of exercise behavior between subjective exercise experience and trait anxiety

Through the mediating effect test, we found that exercise behavior has a partial mediating effect between adolescents’ subjective exercise experience and trait anxiety, which means that the subjective exercise experience can not only directly affect the trait anxiety of adolescents, but also have a significant impact on trait anxiety through the mediating effect of exercise behavior. First, subjective exercise experience was the feeling and experience gained from the existing exercise experience, which could enrich the sports cognitive system, improve the exercise decision-making ability, and help young people to persist in engaging in exercise activities [44]. The experienced philosophy believes that experience was the dependence of cognition and mind on the body, and it was the psychological resource for people to perceive the world, understand the world from the latitude of the body, and use it to change the world [45]. The subjective exercise experience originates from the individual’s perception and understanding of existing exercise and will have a strong impact on future exercise, so those with positive subjective experience often practice physical exercise behavior repeatedly [46]. It could be seen that a good sense of experience will help to enhance the individual’s exercise intention and persistence, and cultivate good exercise habits; otherwise, it will inhibit exercise intention and restrict the implementation of the plan, resulting in a tendency to withdraw [47, 48], which in turn affects the quality of exercise behavior. Second, many studies have shown that physical exercise has a very important role in reducing individual state-trait anxiety [49–51]. The specific performance was that the duration of exercise and the intensity of exercise were significantly negatively correlated with individual trait anxiety, the time of a single exercise was significantly positively correlated with trait anxiety, and the frequency of exercise has no linear relationship with trait anxiety [19]. In the experimental intervention, the improvement effect of exercise on anxiety has also been confirmed [52]. The internal mechanism of this improved benefit may be that physical exercise could promote the production and release of β-endorphins in the body, reduce activities such as adrenaline and cortisol, and stimulate cognitive thinking and emotional cognition, thereby reducing the negative effects such as anxiety in individuals' mood [53]. Therefore, exercise behavior acts as a “bridge” between the subjective exercise experience and trait anxiety.

To deeply examine the mediating effect of exercise behavior, this study used low, moderate, and high exercise amounts to classifying the exercise behavior of adolescents and tested the effect size in different models. The results showed that all three types of exercise behaviors had a partial mediating effect between subjective exercise experience and trait anxiety, but the mediating effect of moderate exercise was the best. Compared with low exercise amount, the subjective exercise experience has a greater effect on moderate and high exercise amount, which can be explained that when adolescents have richer subjective exercise experience, they were more willing to invest more time and energy in physical exercise, so the frequency, time and intensity of exercise were also relatively higher. This was consistent with the view that low-intensity exercisers have a much lower level of positive well-being than moderate and high-intensity exercisers [42]. Meanwhile, compared with low exercise and high exercise, the exercise behavior of moderate exercise amount had the strongest negative predictive effect on trait anxiety, and the mediating effect between subjective exercise experience and trait anxiety was also the strongest. Some studies have pointed out that moderate-intensity exercise activities at least 3 times a week and each exercise duration of no less than 20 min have the best effect on improving trait anxiety [19]. This shows that although a good subjective exercise experience could promote the improvement of individual exercise behavior, it was not that the higher the amount of exercise, the better the effect. When the exercise load exceeds the dose that one can bear, it was likely to bring about negative emotional experiences such as fatigue, irritability, and psychological discomfort, thereby weakening the improvement effect on trait anxiety.

### Limitations

1. Since this study was a cross-sectional study, the results obtained are more subjective and cannot draw deeper causal relationships. Longitudinal empirical studies can be added to future research to better reveal causal associations between variables.
2. This study focused on examining the mediating effect of exercise behaviors with different amounts of exercise between subjective exercise experience and trait anxiety, and more mediating or moderating variables could be explored in the future.

## Conclusions

1. There was no significant gender difference in subjective exercise experience and trait anxiety among adolescents, but boys’ exercise behavior was significantly higher than girls. There was no significant age difference in exercise behavior and trait anxiety, but the subjective exercise experience of 9 to 12 years old students was significantly higher than that of 12 to 15 years old students.
2. The subjective exercise experience of adolescents could directly and positively predict exercise behavior, the exercise behavior could directly and negatively predict trait anxiety, and the subjective exercise experience could directly and negatively predict trait anxiety.
3. The exercise behavior of adolescents played a partial mediating effect between subjective exercise experience and trait anxiety. Among them, compared with low exercise and high exercise, moderate exercise behavior had the strongest mediating effect between subjective exercise experience and trait anxiety.

## Data Availability

The datasets generated and/or analyzed during the current study are not publicly available due to privacy but are available from the corresponding author on reasonable request.
